# Dopaminergic mechanisms in memory consolidation and antidepressant reversal of a chronic mild stress-induced cognitive impairment`

**DOI:** 10.1007/s00213-017-4651-4

**Published:** 2017-05-31

**Authors:** Mariusz Papp, Piotr Gruca, Magdalena Lason-Tyburkiewicz, Ewa Litwa, Monika Niemczyk, Katarzyna Tota-Glowczyk, Paul Willner

**Affiliations:** 1 Institute of Pharmacology, Polish Academy of Sciences, 12 Smetna Street, 31-343 Krakow, Poland; 2 Department of Psychology, Swansea University, Swansea, UK

**Keywords:** Novel object recognition, D1 receptors, D2 receptors, D3 receptors, Prefrontal cortex, Hippocampus, Nucleus accumbens, Chronic mild stress, Venlafaxine, Risperidone, Rat

## Abstract

Cognitive deficits in depression can be modelled using the novel object recognition (NOR) test, performance in which is impaired by chronic mild stress (CMS). We aimed to examine the involvement of mesocorticolimbic DA terminal regions, and to establish the substrate for CMS-induced impairment of NOR and its reversal by chronic antidepressant treatment. In experiments 1 and 2, we examined the effect of infusions into medial PFC, dorsal hippocampus (HPC), and nucleus accumbens (NAc) shell of D1 and D2 antagonists and D3 agonist, which were predicted to impair NOR with a short (1 h) delay, and of D1 and D2 agonists and D3 antagonist, which were predicted to facilitate NOR with a long (24 h) delay. Using optimal doses identified in experiment 2, in experiments 3 and 4, we examined effects on drug-stimulated NOR of CMS and chronic treatment with venlafaxine (VFX) or risperidone (RSP). We found a wide involvement of DA systems in memory for NOR: D1 receptors in PFC, HPC, and NAc; D3 receptors in PFC and HPC; and D2 receptors in PFC. CMS impaired D2- and D3-mediated effects in PFC and HPC; antidepressants rescued those effects in PFC but not HPC. The involvement of DA in NOR is multifaceted, but the effects of CMS and antidepressants are more discrete, involving D2 and D3 receptors in PFC specifically. While raising many difficult questions, these results suggest that the D2 and D3 receptors in the medial PFC may be an important substrate for cognitive deficits in depression and their remediation.

**Electronic supplementary material:**

The online version of this article (doi:10.1007/s00213-017-4651-4) contains supplementary material, which is available to authorized users.

## Introduction

While depression is primarily a disorder of mood and motivation, people who are depressed also present a range of cognitive deficits, including impairments of memory and executive functioning (imperfectly captured in the DSM symptom “Concentration: diminished ability to think or concentrate, or more indecisiveness”: American Psychiatric Association 2013), which are receiving increased attention (Willner 1984; Austin et al. 2001; Carvalho et al. 2014; Belzung et al. 2015; Malhi et al. 2015). It is recognized that these cognitive deficits are associated with functional impairment (Evans et al. 2014; Culpepper 2015) and represent important treatment targets over and above their association with depressed mood, but progress in this area has been limited and the studies that have been conducted suffer from a variety of methodological weaknesses (Papakostas 2015; Miskowiak et al. 2016).

Chronic mild stress (CMS) is a well-validated and widely used animal model of depression, based on the chronic application, over a period of several weeks, of a varying schedule of minor stressors. The effectiveness of CMS is typically established by demonstrating a loss of responsiveness to rewards, which models the core symptom of depression and anhedonia (for reviews *See* Willner 1997a, 2017). However, animals subjected to CMS are also impaired in a variety of spatial and emotional memory tasks (e.g., Song et al. 2012; Cuadrado-Tejedor et al. 2011; Llorente et al. 2011; Gu et al. 2014; Liu et al. 2014; Riaz et al. 2015; Şahin et al. 2015; Tran et al. 2016). These cognitive deficits include impaired performance in the novel object recognition (NOR) test, which, like many other effects of CMS, is rescued by chronic antidepressant treatment (Orsetti et al. 2007; Elizalde et al. 2008; Llorente et al. 2011; Liu et al. 2014; Papp et al. 2016, 2017). In this test, which exploits the animal’s natural tendency to explore novel objects and requires no training, the animal is first exposed to two identical objects: when subsequently re-exposed to one of them alongside a novel object, a greater exploration of the novel object indicates that the familiar object has been remembered (Ennaceur and Delacour 1988). Performance in the NOR is typically good at short test-retest intervals (e.g., 1 h) but absent at longer intervals (e.g., 24 h). This provides a basis for studying both impairment of memory using a 1-h retest (as seen, for example with CMS) and improvement of memory using a 24 h retest. Typically, drugs are administered shortly after the exposure session, to affect specifically the consolidation stage of memory, though other procedures (pre-exposure or pre-retrieval) are also sometimes used.

Drugs affecting many neurotransmitter systems have been reported to influence NOR (*See* e.g., Lyon et al. 2012). We focus here on the dopamine (DA) system, which has received the most attention. Broadly, D1 receptor antagonists and D3 receptor agonists impair NOR, while D1 receptor agonists and D3 receptor antagonists improve NOR (Kamei et al. 2006; Millan et al. 2010, Watson et al. 2011, 2012; de Lima et al. 2011; De Bundel et al. 2013; Rossato et al. 2013; Furini et al. 2014). NOR is also impaired by D2 receptor antagonists ( Watson et al. 2012; França et al. 2015). The major focus of these studies has been the prefrontal cortex (PFC). The region most clearly involved in NOR is the perirhinal cortex (Brown et al. 2012; Balderas et al. 2015). We chose here to focus rather on the medial PFC because of the relevance of this region to depression (Phillips et al. 2003; Hamani et al. 2011; Willner et al. 2013), and because this is the region in which dopaminergic effects on NOR have been described (Watson et al. 2012, De Bundel et al. 2013; Rossato et al. 2013). Additionally, some studies have identified a role for D1 receptors in the dorsal hippocampus (HPC) (De Bundel et al. 2013; Rossato et al. 2013; Furini et al. 2014): for example, a recent study reported improved NOR performance following optogenetic stimulation of DA release in the HPC, which was blocked by a D1 receptor antagonist (Kempadoo et al. 2016).

The aim of this study was to understand whether, and which, DA systems are involved in the impairment of NOR by CMS and its rescue by antidepressant drugs. In light of the literature cited above, while the major focus has been on D1 and D3 receptors in PFC, the effects of CMS and antidepressant on NOR could in principle involve any of D1, D2, and/or D3 receptor systems, in either or both of PFC and HPC. Additionally, while the striatum has received little pharmacological attention in this context, lesion studies suggest a role in NOR for the DA innervation of dorsal striatum (Darvas and Palmiter 2009) and nucleus accumbens (NAc) core (Nelson et al. 2010). The shell of the NAc has not, to our knowledge, been implicated in NOR. We nevertheless elected to target this region, because there is a substantial literature reporting that DA activity in the NAc shell is increased by chronic antidepressant treatment (Papp et al. 1994; Dziedzicka-Wasylewska et al. 1997; Di Chiara et al. 1999).

The NOR test can be used to detect both impairment and enhancement of memory, by testing at either a short post-exposure interval (in our studies, 1 h) when memory is good, or a long post-exposure interval (in our studies, 24 h) when memory is poor. In these experiments, all drugs, irrespective of whether impairment or enhancement of memory was predicted, were administered immediately post-training, and therefore had their effect on the early stages of memory consolidation. The study was in two halves. Experiments 1 and 2 were exploratory studies that aimed to identifying systems (DA receptor subtypes and regions) that displayed bidirectional effects on memory consolidation, using DA receptor-specific drugs that are known to impair (experiment 1) or enhance (experiment 2) NOR. Experiments 3 and 4 aimed to identify the DA systems involved in CMS and antidepressant effects on NOR: because CMS is known to impair NOR, these studies used drugs that enhance NOR.

We first (experiment 1) examined the effects of D1 and D2 antagonists and a D3 agonist injected into medial PFC, dorsal HPC, or NAc shell. These drugs were all predicted to impair NOR, so experiments were conducted with a brief (1 h) delay. A failure to see an impairment for a particular combination of drug and brain region would imply that this mechanism does not play a role in spontaneous NOR. Next (experiment 2), we performed the opposite set of experiments, using D1 and D2 agonists and a D3 antagonist. These drugs were predicted to enhance NOR, so experiments were conducted with a long (24 h) delay. A failure to see an enhancement for a particular combination of drug and brain region would imply that this mechanism is not a substrate for CMS to impair NOR (assuming that the mechanism by which receptor-specific drugs enhance NOR is a summation of the effects of the drug and of endogenous DA to produce a greater signal at the same receptor, leading to the formation of a stronger and therefore longer-lasting memory trace). To our knowledge, this is the first study to examine in parallel the involvement of multiple DA receptor subtypes in multiple brain regions in both early impairment and late enhancement of NOR. In experiment 3, we examined the effect of CMS on each of the seven drug/region combinations where facilitatory effects had been observed in experiment 2. Finally (experiment 4), we studied the rescue of CMS-impaired NOR by chronic antidepressant treatment.

## Methods

### Subjects

Male Wistar rats (Charles River, Germany), weighing 200–230 g at the start, were used in all experiments. Except as described below, they were housed singly with free access to food and water, and maintained on a 12-h light/dark cycle (lights on at 08.00 h) in conditions of constant temperature (22 ± 20 °C) and humidity (50 ± 5%). Group sizes were *n* = 8 in experiments 1 and 2, *n* = 10 in experiment 3, and *n* = 6 in experiment 4. All procedures used conformed to the rules and principles of EEC Directive 86/609 and were approved by the Bioethical Committee at the Institute of Pharmacology, Polish Academy of Sciences, Krakow, Poland.

### Novel object recognition test

NOR was tested in a non-transparent open field (100 cm in diameter, 35 cm high, with the floor divided by painted lines into 16-cm squares). After 10-min adaptation sessions on two successive days, each animal was allowed to explore two identical objects (white cylinders, 7 cm in diameter, 11-cm high) for the time required to complete 20 s of exploration of both objects (T1 session). In a retention trial conducted 1 h (T2 session) or 24 h (T3 session) later, one of the objects presented previously was replaced by a novel object (black prism, 5-cm wide, 14-cm high). Rats were returned to the open field for 5 min and the duration of exploration of each object (defined as sitting in close proximity to the objects, sniffing, or touching them) was measured by a trained observer who was blind to drug treatment. A recognition index was calculated according to the formula: time of novel object exploration minus time of familiar object exploration, divided by total exploration time (novel plus familiar objects), multiplied by 100 (Akkerman et al. 2012). During NOR sessions, the number of line crossings was recorded as a measure of locomotor activity.

### Study design

The study comprised two series of experiments. In the first series (experiments 1 and 2), the effects of a range of doses of D1, D2, and D3 agonists and antagonists were evaluated in the NOR test in control, non-stressed animals, while the second series (experiments 3 and 4) examined the effect of CMS on drug-enhanced NOR and the reversal of CMS effects by an antidepressant, venlafaxine (VFX), and risperidone (RSP), an atypical antipsychotic that is also used clinically for depression.

Experiment 1 tested the effects of drugs that were predicted to impair NOR: the D1 antagonist SCH23390 (0.5–3 μg) (Cussac et al. 2004), the D2 antagonist L-741,626 (0.5–2.5 μg) (Bristow et al. 1998), and the D3 agonist 7-OH-DPAT (0.1–10 μg) (Lévesque et al. 1992). These drugs were injected into either the PFC, HPC, or NAc immediately after the initial object exposure (T1 session), and their effects were tested 1 h later (T2 session), when animals typically show good NOR performance.

Experiment 2 tested the effects of drugs that were predicted to improve NOR: the D1 agonist SKF 81297 (0.05–0.75 μg) (Arnt et al. 1988), the D2 agonist quinpirole (0.1–5 μg) (Lokhandwala and Steenberg 1984), and the D3 antagonist SB-277,011 (0.1–1 μg) (Reavill et al. 2000). These drugs were injected into either the PFC, HPC, or NAc immediately after the initial object exposure (T1 session), and their effects were tested 24 h later (T3 session), when animals typically show poor NOR performance.

Enhancement of NOR was seen in seven of the nine conditions tested in experiment 2. For experiment 3, the optimally effective dose was tested in control animals and animals subjected to CMS. CMS was predicted to impair drug-enhanced NOR performance, so tests were conducted 24 h post-exposure (T3).

Impairment of NOR by CMS was seen in four of the seven conditions tested in experiment 3, reflecting D2- and D3-mediated (but not D1) effects in the PFC and HPC. In experiment 4, D1-, D2-, and D3-mediated effects in the PFC and HPC were each retested in two cohorts of animals treated chronically with either RSP (1 ml/kg i.p.) or VFX (10 mg/kg i.p.), respectively, which were predicted to reverse the effects of CMS.

### Chronic mild stress (CMS) procedure

In CMS experiments, after 3 weeks of habituation to laboratory and housing conditions, the animals were trained to consume a 1% sucrose solution in eight baseline tests conducted once weekly in the home cage. After 14-h food and water deprivation, the animals were presented with a freshly prepared 1% sucrose solution for 1 h. Sucrose intake was calculated by weighing bottles before and after the test. Subsequently, sucrose consumption was monitored once weekly, under similar conditions, until the end of the study.

On the basis of their intakes in the final baseline test, the animals were divided into two matched groups: control (CON) and stressed (CMS). The control animals were housed in separate rooms and were deprived of food and water for 14 h before each sucrose test, but otherwise food and water were available freely available. Each week of the stress regime consisted of two periods of food or water deprivation, two periods of 45° cage tilt, two periods of intermittent illumination (light on and off every 2 h), two periods of soiled cage (250-ml water in sawdust bedding), one period of paired housing, two periods of low-intensity stroboscopic illumination (150 flashes/min), and three periods of no stress. The duration of all stressors was 10–14 h and they were applied individually and continuously, day and night. In most stressed animals (approx. 80%), this procedure causes a gradual decrease in the consumption of the sucrose solution to approximately 40% of the pre-stress values (*See* Papp 2012).

In experiment 4, treatment with RSP and VFX commenced after 2 weeks of stress and continued to the end of the experiment. Daily injections of vehicle, RSP, or VFX were administered mid-morning, and preceded weekly sucrose intake tests by approximately 24 h.

All animals (CON and CMS) were implanted with bilateral cannulae, aimed at the mPFC, dHPC, or NAS, after 5 weeks of CMS. One (experiment 3) or two (experiment 4) weeks later a final sucrose test was conducted, followed by the NOR procedure, as described above.

### Stereotaxic surgery and intracranial injection procedure

Animals were anesthetized with ketamine (80 mg/kg) and xylazine (5 mg/kg) and placed in a stereotaxic apparatus (Stoelting Co., Wood Dale, IL, USA). Bilateral stainless steel guide cannulae (0.6-mm external diameter) were inserted into the medial PFC (AP +3.0 mm, L +/−0.7 mm, DV −2.8 mm from bregma), the dorsal HPC (AP −4.2 mm, L +/−2.8 mm, DV −1.9 mm from bregma), or the shell of the NAc (AP +1.7 mm, L +/−1.0 mm, DV −6.3 mm from bregma) according to the atlas of Paxinos and Watson (1994). The cannulae were fixed to the skull with stainless screws and dental cement (Adhesor Carboline, SpofaDental, Jicin, Czech Republic). Stainless steel obturators were placed in the guide cannulae to prevent occlusion. After surgery, the rats were allowed to recover for at least 7 days before the start of behavioral testing.

On the injection day, two stainless steel internal cannulae (0.4-mm external diameter, extending 0.7 mm below the guide cannulae) were inserted into the guide cannulae and 0.5 μl of solution was infused bilaterally. Infusions were made over 1 min, using an infusion pump and 2-μl Hamilton syringes. All infusions were made bilaterally immediately following the exposure session (T1). The internal cannulae were left in place for 1 min to avoid backflow of the infusion.

At the end of the experiment, the correct placement of cannulae was verified in frozen coronal sections of brains cut throughout the target areas with digital pictures taken to visualize the cannula routes and the injection sites (Supplementary Fig. S1). Animals in which the cannula tips were found to be outside the target areas, which amounted to a total of 3.4% overall, were excluded from the data analysis.

### Drugs

L-741,626 was dissolved in 5% DMSO and 5% Cremophor EL (Loiseau and Millan 2009); all other compounds were dissolved in 0.9% sterile saline. The drugs used for intracranial injections were purchased from Tocris Bioscience, Avonmouth, Bristol, UK, and doses were selected on the basis of data in the literature showing their efficacy in modulating performance in various cognitive tasks (da Silva et al. 2012; Loiseau and Millan 2009; Nagai et al. 2007; Watson et al. 2012; Wilkerson and Levin 1999), and on our unpublished preliminary studies. VFX HCl and RSP were purchased at Sequoia Research Products Ltd., Pangbourne, UK. The doses were selected on the basis of our previous studies showing their efficacy against the CMS-induced deficit in sucrose consumption (Marston et al. 2011; Millan et al. 2001).

### Statistical analysis

Data from NOR tests (recognition index, exploration time, and locomotor activity) were analyzed by one-way ANOVA (dose) in experiments 1 and 2, and Duncan’s test was used for post hoc testing of drug effects relative to vehicle treatment. Two-way ANOVA (challenge drug × CMS) was used in experiment 3. In experiment 4, a four-way ANOVA was applied to the data from each injection site: challenge drug (vehicle/quinpirole/SB-277,011) × stress (CON/CMS) × antidepressant treatment (present/absent) × treatment drug (RSP/VFX).

Sucrose intake was analyzed by two-way ANOVA (CMS × weeks: experiment 3) or three-way ANOVA (drugs × CMS × weeks: experiment 4). All data were entered into the analyses: results are presented for the baseline and final tests (experiment 3) and the test preceding the start of drug treatment (experiment 4).

The results of most statistical analyses are presented in online Supplementary tables.

## Results

### Experiment 1

**Fig. 1:**
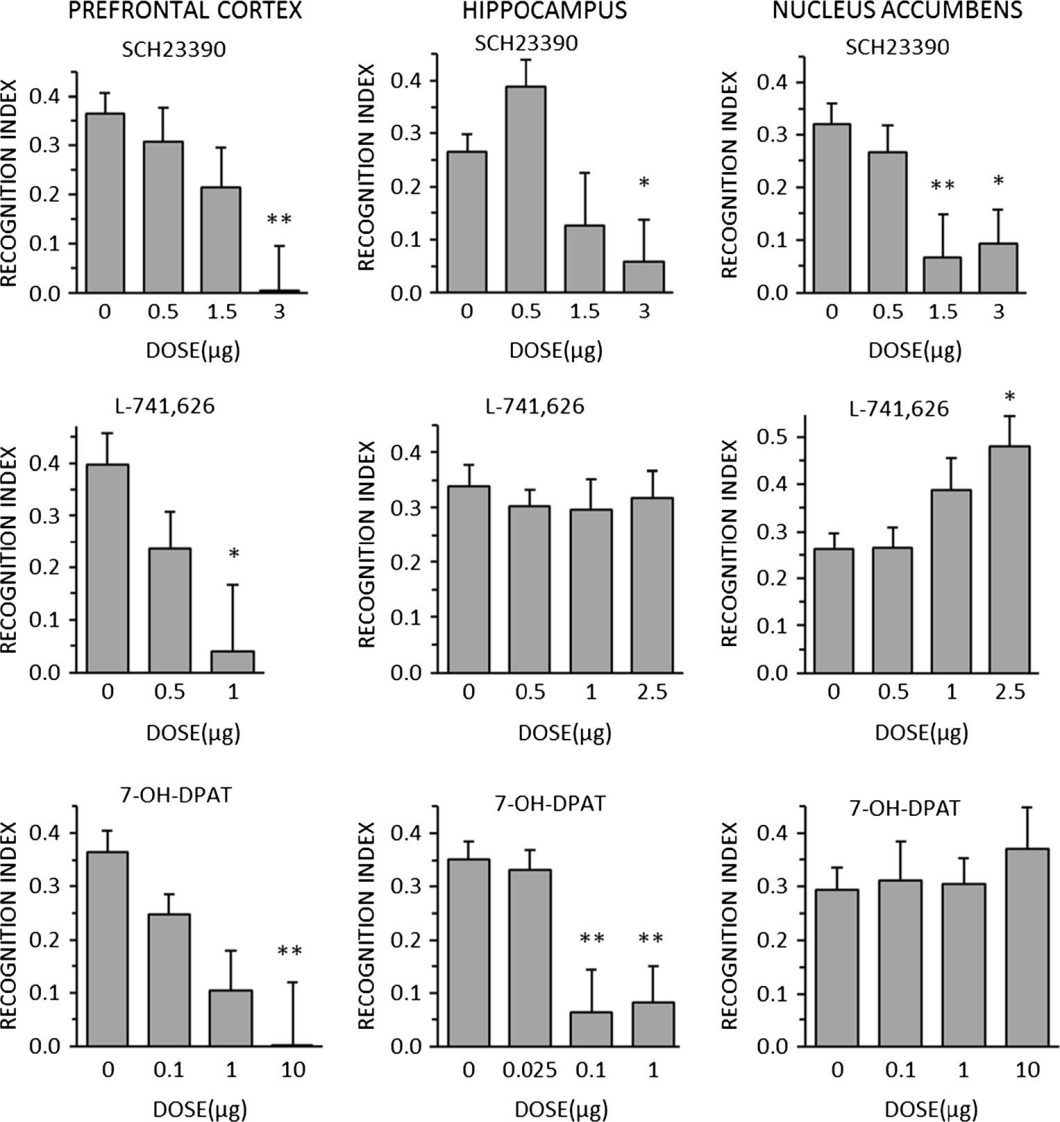
Effects of the D1 antagonist SCH23390 (*top row*), the D2 antagonist L-741,626 (*middle row*), and the D3 agonist 7-OH-DPAT (*bottom row*), injected bilaterally into the medial PFC (*left column*), the dorsal HPC (*middle column*), or the shell of the NAc (*right column*), on novel object recognition 1 h post-training. Data are shown as mean + SEM. *p < 0.05, **p < 0.01 relative to vehicle (0 μg) treatment

The effects of SCH23390, L-741,626, and 7-OH-DPAT on NOR at T2 (1 h post-training) are shown in Fig. 1. All three drugs impaired NOR when injected into the PFC. Similar effects were seen when SCH23390 and 7-OH-DPAT, but not L-741,626, were injected into the HPC. Only SCH23390 impaired NOR when injected into the NAc: 7-OH-DPAT was without effect, while L-741,626 significantly improved NOR. However, it should be noted that the L-741,626 experiment had the lowest control level of NOR and the significance of the effect was marginal (*p* = 0.041). In order to test the reality of this effect, an additional experiment was performed in which L-741,626 was injected into the NAc prior to a T3 test (24 h post-training, when NOR is minimal). There was no increase in NOR (mean ± SEM: VEH, 0.05 ± 0.06; L741626, 0.11 ± 0.04).

The ANOVA results are shown in Supplementary Table S1, which also shows the results for exploration and locomotor activity. There were no significant effects on exploration, and just one marginal (*p* = 0.041) increase in locomotor activity (at the lower dose of L-741,626). A significant effect in 1/18 analyses would be expected by chance alone.

### Experiment 2

**Fig. 2:**
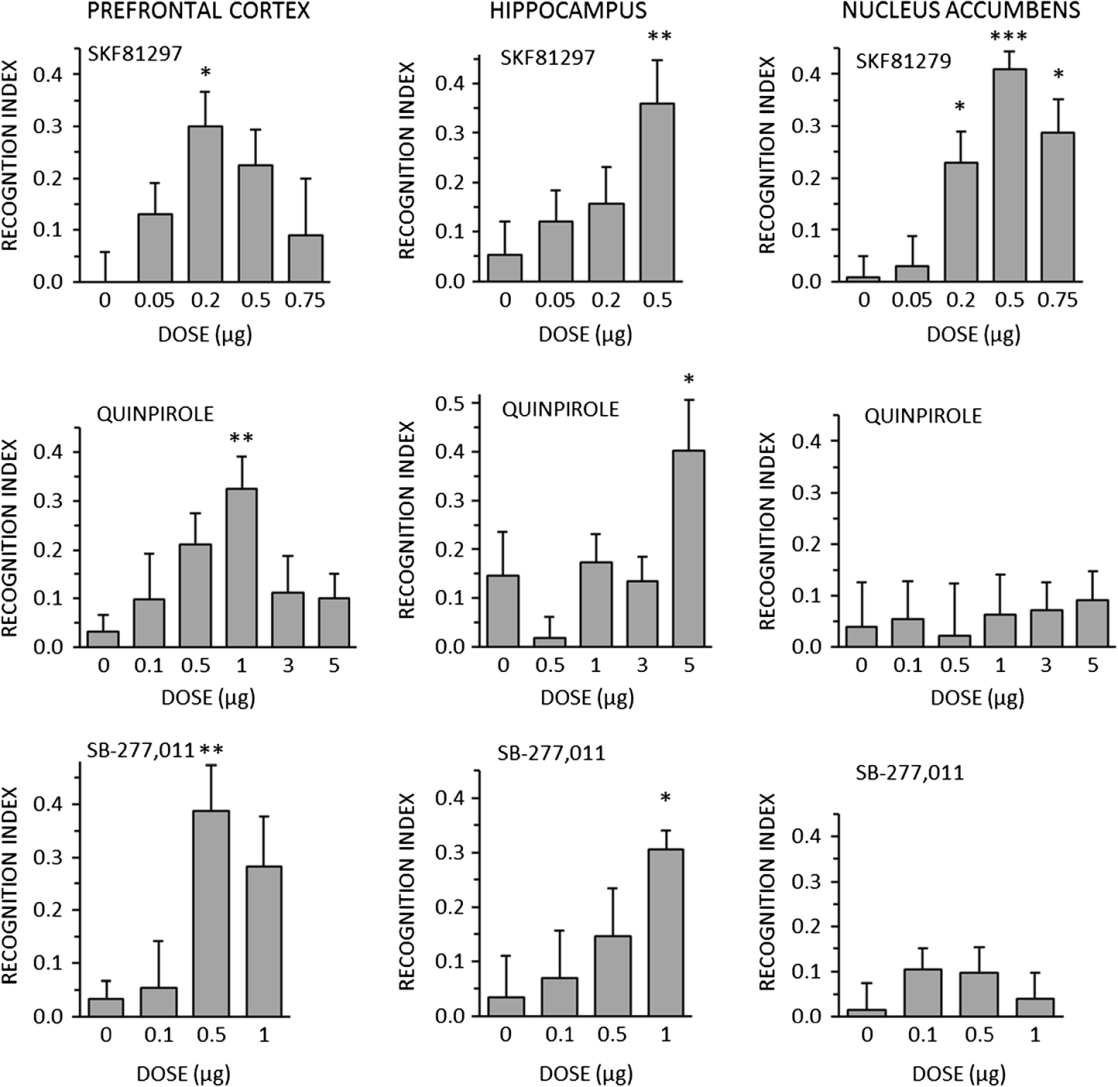
Effects of the D1 agonist SKF81297 (*top row*), the D2 agonist quinpirole (*middle row*), and the D3 antagonist SB-277,011 (*bottom row*), injected bilaterally into the medial PFC (*left column*), the dorsal HPC (*middle column*), or the shell of the NAc (*right column*), on novel object recognition 24 h post-training. Data are shown as mean + SEM. *p < 0.05, **p < 0.01, ***p < 0.001 relative to vehicle (0 μg) treatment

The effects of SKF81297, quinpirole, and SB-277,011 on NOR at T3 (24 h post-training) are shown in Fig. 2. All three drugs improved NOR at one or more doses when injected into the PFC or HPC, but only SKF81279 enhanced NOR when injected into the NAc. Dose-response functions were bell-shaped for all four conditions where the dose range tested included doses above the lowest active dose.

The ANOVA results are shown in Supplementary Table S1, which also shows the results for exploration and locomotor activity. There were marginally significant effects of SB-277,011 in the HPC on exploration and of quinpirole in the NAc on locomotor activity, but in neither case did any individual drug dose differ significantly from vehicle.

### Experiment 3

Experiment 3 was a partial replication of experiment 2, with the omission of the two ineffective challenges (quinpirole and SB-277,011 in the NAc). In addition, all groups were tested under control conditions and after CMS. CMS significantly decreased sucrose intake (*F*(1,135) = 219.95, *p* < 0.001); the effect was present after a single week of CMS (mean ± SEM (ml): CON, 10.72 ± 0.33; CMS, 5.33 ± 0.33), and persisted through the final sucrose intake test, following cannula implantation and immediately preceding the NOR test (mean ± SEM (ml): CON, 11.20 ± 0.43; CMS, 6.14 ± 0.44).

**Fig. 3:**
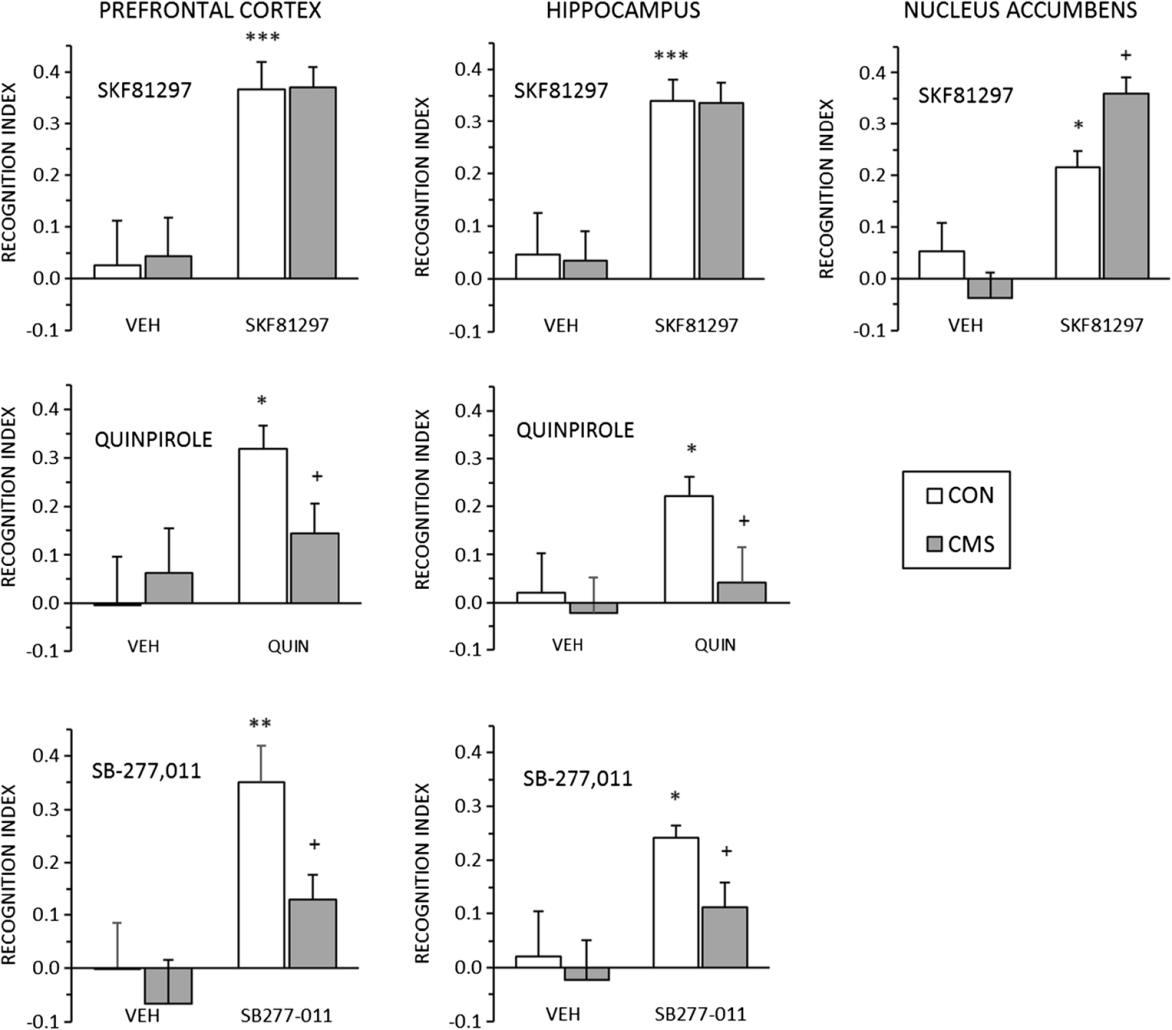
Effects of the D1 agonist SKF81297 (*top row*) injected into PFC, HPC, or NAc, the D2 agonist quinpirole (*middle row*) injected into PFC and HPC, and the D3 antagonist SB-288,011 (*bottom row*) injected into PFC and HPC. Drug doses (cf. Fig. 2) were PFC: SKF81297 0.2 μg, quinpirole 1 μg, SB-277,011 0.5 μg; HPC: SKF81297 0.5 μg, quinpirole 5 μg, SB-277,011 1 μg; NAc: SKF81297 0.5 μg. Data are shown as mean + SEM. *p < 0.05, **p < 0.01, ***p < 0.001 for challenge drug vs. vehicle in control groups; ^+^ *p* < 0.05 for CON vs CMS in challenge drug groups

All seven agonist (D1, D2) and antagonist (D3) challenges increased NOR, replicating the effects seen in experiment 2 (Fig. 3). At all three sites tested (PFC, HPC and NAc), CMS failed to block the effect of SKF81297 (SKF81297 actually increased NOR when injected into the NAc of stressed animals, but this was from a lower control baseline than seen with SKF81297 injections either in the PFC or HPC in experiment 3, or in the NAc in experiment 2). However, in contrast to the lack of interaction with D1-mediated effects, CMS did significantly block the effects of both quinpirole and SB-277,011, in both PFC and HPC (Fig. 3).

The ANOVA results are shown in Supplementary Table S2. The drug × CMS interaction terms were non-significant, but planned comparisons showed that all challenges increased NOR (confirming the results of experiment 2) and that CMS did block the effects of quinpirole and SB-277,011 but not those of SKF81297. Analyses of the results for exploration and locomotor activity found no significant main effects (results not shown) or interactions (Supplementary Table S2).

### Experiment 4

CMS decreased sucrose intake, and the effect was completely reversed by chronic treatment with RSP or VFX (Table 1), as confirmed by a significant drugs × CMS × weeks interaction (*F*(12,1260) = 7.96, *p* < 0.001).

**Table 1:** Effects of CMS and antidepressant treatment on sucrose intake^a^

Week	Vehicle		Risperidone (1 mg/kg)		Venlafaxine (10 mg/kg)	
	CON	CMS	CON	CMS	CON	CMS
0	12.59 (±0.78)	12.06 (±0.41)	12.68 (±0.73)	12.00 (±0.37)	11.91 (±0.71)	13.56 (±0.46)
2	13.27 (±0.95)	6.25 (±0.48)***	12.77 (±0.71)	6.45 (±0.33)***	12.45 (±0.61)	7.20 (±0.39)***
7	13.25 (±0.69)	5.35 (±0.50)***	12.78 (±0.80)	12.16 (±0.35)	12.61 (±0.74)	13.12 (±0.52)

^a^Data are shown (mean ± SEM sucrose consumed in 1-h weekly tests) for weeks 0 (pre-stress), 2 (start of daily treatment with vehicle, risperidone, or venlafaxine), and 7 (final sucrose intake test after cannula implantation and immediately preceding the NOR test). ***p < 0.001 relative to weeks 0 and/or 7.

**Fig. 4:**
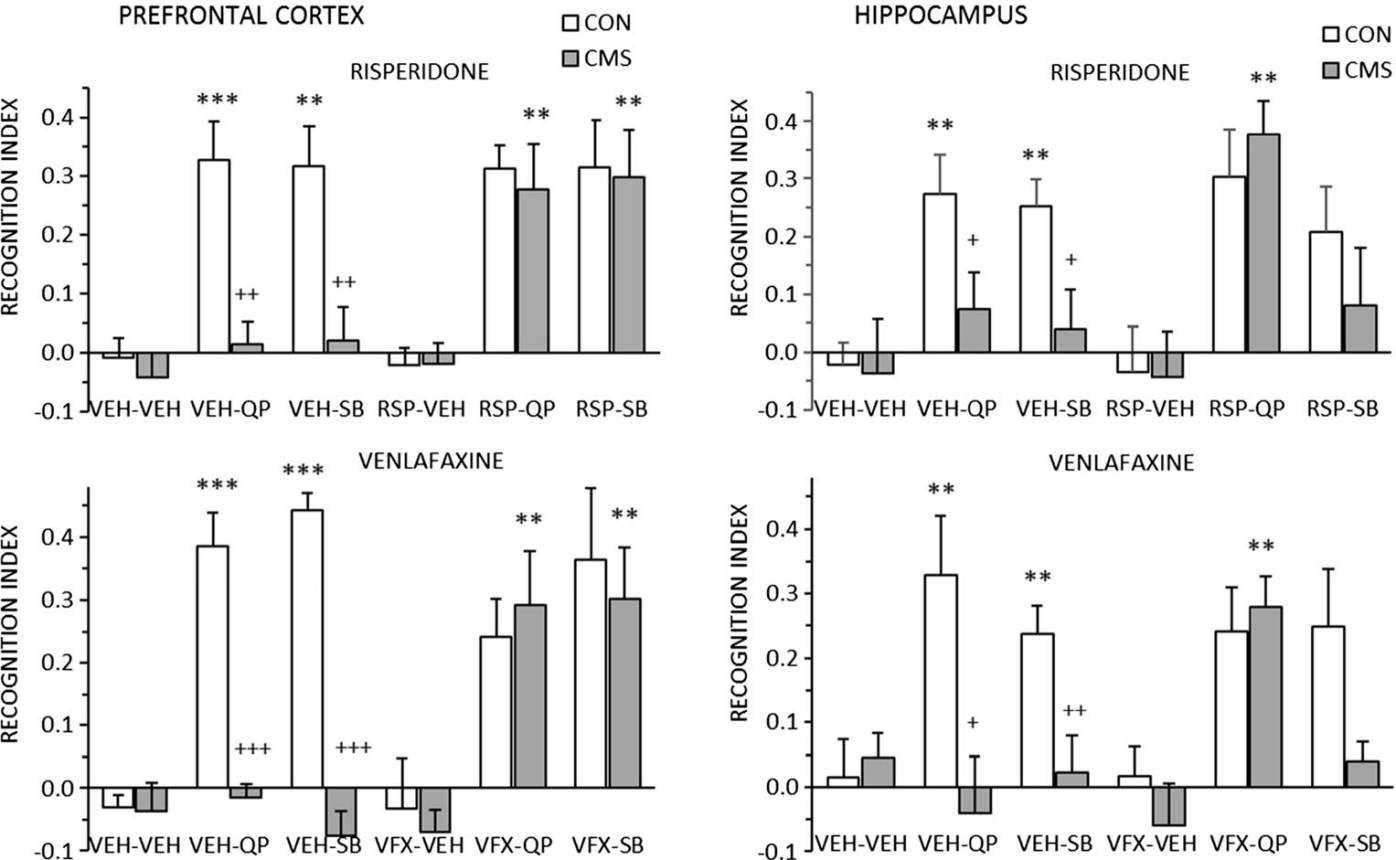
Control (*CON*, *white bars*) and stressed (*CMS*, *gray bars*) animals were challenged with the D2 agonist quinpirole (*QP*) or the D3 antagonist SB277-011 (*SB*) injected into the PFC (*left panels*) or HPC (*right panels*), after chronic (5 weeks) pre-treatment with vehicle (*VEH*), risperidone (*RSP* 1 mg/kg i.p., *upper panels*), or venlafaxine (*VF* 10 mg/kg i.p., *lower panels*). QP and SB were administered at the same doses as shown in Fig. 4. Data are shown as mean + SEM. **p < 0.01, ***p < 0.001 relative to the respective vehicle-treated groups; ^+^ *p* < 0.05, ^++^ *p* < 0.01, ^+++^ *p* < 0.001 for CON vs. CMS. Significance levels are only shown for comparisons relevant to the hypotheses under test: stimulation of NOR by QP and SB; blockade of those effects by CMS; and rescue by RSP or VFX

Experiment 4 replicated the results of experiment 2 (increased NOR following quinpirole or SB-277,011 in both PFC and HPC) and experiment 3 (blockade of all four effects by CMS). Chronic pre-treatment with both RSP and VFX reversed the blockade by CMS of quinpirole effects in both PFC and HPC, and of the effects of SB-277,011 in the PFC. However, neither drug rescued the blockade by CMS of the effects of SB-277,011 in the HPC (Fig. 4).

The analyses of variance (Supplementary Table S3) confirmed significant stress × challenge × antidepressant treatment interactions for both PFC (*p* = 0.003) and HPC (*p* = 0.018), but there were no significant effects of treatment drug, indicating that the effects of RSP and VFX did not differ significantly. In order to explore the interactions, two-way ANOVAs (stress × treatment) were conducted for each of the quinpirole and SB-277,011 challenges in PFC and HPC. The stress × treatment interactions were highly significant (*p* < 0.001) for quinpirole at both sites and for SB-277,011 in the PFC; however, for SB-277,011 in the HPC, both the main effect of treatment and the stress × treatment interaction were non-significant (*F* < 1).

Experiment 4 also replicated the ineffectiveness of CMS to inhibit SKF 82197-stimulated NOR in the PFC and HPC (mean ± SEM NOR, *n* = 12/group: PFC, CON 0.34 ± 0.03, CMS 0.33 ± 0.04; HPC, CON 0.25 ± 0.02, CMS 0.30 ± 0.03). Predictably, given this lack of effect of CMS, there was also no effect of VFX or RSP (results not shown).

## Discussion

The results of these studies, as summarized in Table 2, were as follows:

**Table 2:** Summary of results

	PFC			HPC			NAc		
	D1	D2	D3	D1	D2	D3	D1	D2	D3
Early (1 h) impairment^a^	Yes	Yes	Yes	Yes	No	Yes	Yes	No	No
Late (24 h) enhancement^b^	Yes	Yes	Yes	Yes	Yes	Yes	Yes	No	No
Impairment (24 h) by CMS^c^	No	Yes	Yes	No	Yes	Yes	No	NT	NT
Restoration by VFX and RISP^c^	NT	Yes	Yes	NT	Yes	No	NT	NT	NT

^a^Drugs used: D1 antagonist (SCH 233390), D2 antagonist (L-471,626), D3 agonist (7-OH-DPAT)

^b^Drugs used: D1 agonist (SKF 81297), D2 agonist (quinpirole), D3 antagonist (SB-277,011)

^c^ *NT* not tested

(i) In all three regions (PFC, HPC, and NAc shell), NOR was dose-dependently impaired by the D1 antagonist SCH 233390 and enhanced by the D1 agonist SKF 81297. However, CMS did not impair D1-stimulated NOR in any of PFC, HPC, or NAc.

(ii) The D2 agonist quinpirole and the D3 antagonist SB-277,011 also dose-dependently enhanced NOR when injected into the PFC or HPC (but not the NAc), but whereas the both the D3 antagonist 7-OH-DPAT and the D2 antagonist L-741,626 impaired NOR when injected into the PFC, only 7-OH-DPAT (but not L-741-626) had this effect in the HPC.

(iii) CMS impaired both D2- and D3-stimulated NOR in both PFC and HPC. Antidepressant treatment with either VFX or RSP restored both of these effects in PFC, but only the D2 effect in HPC. Antidepressant treatment did not improve NOR following CMS in animals not receiving D2/D3 stimulation, indicating that the effect is not an improvement of memory consolidation per se, but rather a reversal of CMS effects on D2/D3 receptors specifically.

(iv) In all experiments, the results can be assumed to reflect differential effects on memory consolidation, because drug effects on exploration and locomotor activity during the test session were minimal or absent. The use of post-exposure drug administration also rules out state-dependent learning and differences in attention or motivation during object exposure as explanations of the results.

### D1 receptors

Systemic administration of D1 receptor agonists is known to improve performance in memory and other cognitive tasks, while D1 receptor antagonists impair cognitive performance (e.g., Hotte et al. 2005, 2006; Nikiforuk 2012; Lejeune et al. 2013). The PFC is known to play an important role in these effects, since cognitive performance is typically impaired by D1 antagonists and improved by moderate doses of D1 agonists infused locally into the PFC (Chudasama and Robbins 2006; Puig et al. 2014). Higher doses of D1 agonists typically impair cognition: an inverted U-shaped dose-response relationship between D1 receptor stimulation in PFC and cognitive performance is seen in both animal (Levy 2009) and human (Takahashi et al. 2012) studies. The observed impairment of NOR by the D1 antagonist SCH23390 and dose-dependent facilitation of NOR by the D1 agonist SKF 81297, when infused into the PFC, are consistent with this general picture, and with previous studies in the NOR paradigm specifically (Kamei et al. 2006; Watson et al. 2012; De Bundel et al. 2013; Rossato et al. 2013; Furini et al. 2014).

D1 (or possibly D5) receptors in the HPC are also known to play a role in cognition. Studies have focused largely on spatial memory (Hersi et al. 1995; Seamans et al. 1998; Xing et al. 2010; da Silva et al. 2012), but there are also reports of performance in the NOR test being impaired by the D1 antagonist SCH 23390 and improved by the D1 agonist SKF 81297 infused into the dorsal HPC (De Bundel et al. 2013; Furini et al. 2014; Kempadoo et al. 2016). The effects of SCH 23390 and SKF 81297 observed in the present study are consistent with these earlier observations (there is also a negative report with SCH 23390 (Rossato et al. 2013), but the study used a single low dose, 1.5 μg/side, that was also ineffective here). Pro-cognitive effects of D1 agonists have also been reported, in other tests, following infusion into the shell of the NAc (Pezze et al. 2007; Loiseau and Millan 2009). To our knowledge, the effects on NOR of D1 agonists and antagonists infused into the NAc shell have not previously been studied. Our data suggest that while there might be minor differences in potency, the effects of both SCH 23390 and SKF 81297 were broadly equivalent when infused into any of PFC, HPC, or NAc shell, suggesting that the consolidation of memory for objects may involve D1-mediated DA signaling in all three regions.

Be this as it may, the results are unequivocal that CMS had no effect on D1-stimulated facilitation of NOR in any of the three areas studied, indicating that a decrease in transmission at D1 receptors is not involved in the impairment of NOR by CMS. This is consistent with studies reporting that CMS and other chronic stress procedures either have minimal effects on D1 receptors (Papp et al. 1994; Dziedzicka-Wasylewska et al. 1997; Lucas et al. 2004 Delis et al. 2015; Jin et al. 2015), or increase D1 receptor density in the NAc specifically (Ossowska et al. 2001; Scheggi et al. 2002). Given the clear evidence that D1 receptors were not involved in the impairment of NOR by CMS, it is entirely unsurprising that there was also no effect of antidepressant treatment on D1-stimulated facilitation of NOR.

### D2 receptors

Systemic administration of D2 receptor antagonists has also been reported to impair performance in certain cognitive tasks (Floresco et al. 2006; Millan et al. 2007; Puig et al. 2014), including NOR (Braszko 2006; Watson et al. 2011; França et al. 2015, França et al. 2016). This effect has been reported for the D2 antagonist L-741,626 administered within the PFC (Watson et al. 2012); we are not aware of studies targeting other brain regions. Here, we confirmed the impairment of NOR by L-741,626 injected into the PFC, and also report that this effect appears to be specific to the PFC, because L-741,626 did not impair NOR when injected into the dorsal HPC or NAc shell (at doses more than twice the effective dose in the PFC).

While less studied than the effects of D1 agonists, there are some isolated reports of memory facilitation by the D2 receptor agonist quinpirole (Fujishiro et al. 2005; Cardoso-Cruz et al. 2014). We are aware of only two studies testing the effect of quinpirole on NOR, both of which were negative (de Lima et al. 2011; Rossato et al. 2013). However, in both cases, quinpirole was administered, systemically, to animals displaying good retention of NOR; that is, under conditions where facilitation would be difficult to demonstrate. Here, we report that when tested in animals showing poor retention (24 h post-exposure under our experimental conditions), quinpirole facilitated retention of NOR when injected into either the PFC or the HPC, but not when injected into the NAc. However, the significance of the effect of quinpirole in the HPC is questionable. First, this effect was seen only at the highest dose of quinpirole (5 μg/side), five times the effective dose in the PFC, which could indicate a loss of specificity. Second, and more important, unlike the situation in the PFC, there was no corresponding impairment of NOR by D2 antagonism in the HPC. This implies that the consolidation of memory for NOR does not require dopaminergic stimulation of D2 receptors in the HPC. Hence, it appears that, while of pharmacological interest, the effect of quinpirole to facilitate NOR when injected into the HPC is non-physiological.

Both of these effects of quinpirole, in PFC and HPC, were absent in animals exposed to CMS. However, the effect of quinpirole in the HPC is not relevant to an understanding of the impairment of NOR by CMS in the absence of stimulatory drug effects, because the absence of a D2 antagonist effect in the HPC implies that D2 receptors in the HPC do not play a role in unstimulated NOR. Using the same logic, while chronic treatment of CMS animals with the antidepressants VFX and RSP restored the stimulatory effect of quinpirole in both the PFC and HPC, the former effect is relevant to the mechanism of action of CMS and antidepressants, but the latter effect is not. We conclude that, in the absence of dopaminergic stimulation, both the impairment of quinpirole-stimulated NOR by CMS and the rescue of NOR by chronic antidepressant treatment involve D2 receptors in the PFC specifically.

D2 receptors are of interest in this context because there is clinical evidence that D2 agonists are effective as antidepressants (Willner 1997b; Gershon et al. 2007) and specific D2 receptor antagonists have been shown to reverse the action of antidepressant drugs both in the CMS model (Sampson et al. 1991; Muscat et al. 1992; Zebrowska-Lupina et al. 1992; Papp et al. 1994; D’Aquila et al. 1997) and in depressed patients (Willner et al. 2005). However, these effects have been attributed to an upregulation of D2 receptors in the NAc, a well-established effect of chronic antidepressant treatment (Willner 1997b; Gershon et al. 2007) that is observed, inter alia, both in rats following CMS (Papp et al. 1994; Dziedzicka-Wasylewska et al. 1997) and in depressed patients (Bowden et al. 1997; Larisch et al. 1997; D’haenen et al. 1999; Klimke et al. 1999).

The medial PFC is now the major focus of interest for studies of depression, and increasingly for studies of antidepressant action (Ernst and Paulus 2005; Mayberg 2009; Hamani et al. 2011; Willner et al. 2013). There has been relatively little analysis of the role of DA, and specifically, D2 receptors, in this region. However, there is evidence that CMS decreases reward-related DA release in the PFC (Di Chiara et al. 1999), and a recent study reported that D2-mediated transmission to glutamatergic medial PFC neurons was responsible for antidepressant rescue of anhedonic and other depression-relevant behaviors in chronically stressed mice (Seo et al. 2016). This mechanism could plausibly underlie the D2-mediated effects on NOR reported here.

### D3 receptors

A number of recent studies have reported that NOR is improved by systemic administration of D3 antagonists, both under control conditions (Watson et al. 2011) and in pharmacological models relevant to schizophrenia or ADHD (Barth et al. 2013; Neill et al. 2016; Watson et al. 2016; Sun et al. 2016); impairment of NOR by a D3 agonist has also been reported. Similar facilitatory effects of D3 antagonists and inhibitory effects of D3 agonists, following systemic administration, have been reported in other cognitive tasks (Millan et al. 2010; Nakajima et al. 2013; Sokoloff and Le Foll 2017). A facilitatory effect on both NOR and social recognition was also seen with direct administration of D3 antagonists within the medial PFC (Loiseau and Millan 2009; Watson et al. 2012). Here, we confirm the facilitatory effect of D3 antagonism in the medial PFC, and report the opposite effect, an impairment of NOR by a D3 agonist in the medial PFC; and we report that the same two effects were seen in the dorsal HPC (but not in the NAc). To our knowledge, D3-mediated effects on NOR have not previously been studied in the HPC. However, it has been reported that the D3 antagonists SB-277011A and U99194A decreased tissue levels of acetylcholine (indicating increased acetylcholine release) to a similar extent in PFC and HPC (Barth et al. 2013), contrary to earlier reports that suggested a specific action within the PFC (Nakajima et al. 2013). Other potential mechanisms of action include interactions with NMDA glutamate receptors in PFC (Sokoloff et al. 2013; Sokoloff and Le Foll 2017) and with cAMP-PKA-CREB signaling in HPC (Xing et al. 2010).

D3 receptors in the PFC are located both post-synaptically on glutamatergic neurons and pre-synaptically on DA terminals, but a pre-synaptic inhibitory action on DA release appears to predominate, because D3 knockout animals have extracellular levels of DA that are double those seen in wild-type animals (Le Foll et al. 2005; Song et al. 2012). In several studies utilizing the forced swim test, these animals have been reported to display an antidepressant-like phenotype: relative to wild-type animals they were resistant to effects of repeated stress (Xing et al. 2013) and more sensitive to antidepressant drugs (Leggio et al. 2008); and both a knockout of D3 receptors and the D3 antagonist SB 277,011 prevented the effect of adolescent stress to increase immobility in adult animals (Seo and Kuzhikandathil 2015). Consistent with this picture, an antidepressant effect of the D3 antagonist/partial agonist cariprazine has been reported both in the CMS model (Papp et al. 2014), and in randomized controlled trials of monotherapy for bipolar depression and adjunctive treatment in major depression (Durgam et al. 2016). The D3-preferring agonist pramipexole is also an effective antidepressant in the CMS model (Willner et al. 1994) and clinically (Corrigan et al. 2000; Cassano et al. 2004; Zarate et al. 2004). It is uncertain whether these effects of pramipexole are mediated primarily via D3 or D2 receptors. However, a recent study reported that chronic treatment with pramipexole caused an increase in extracellular DA levels—similar to the effect of D3 antagonists—that was absent in D3 knockout animals, but preserved in D2 knockouts (Castro-Hernández et al. 2015), suggesting a D3-mediated effect.

To our knowledge, there are no published reports of the effects of CMS or other chronic stress procedures on the expression or function of D3 receptors. Consequently, no mechanism suggests itself for the abolition by CMS of the stimulatory effect on NOR of the D3 antagonist SB-277,011. There is a similar vacuum with respect to the effects of antidepressant treatment to reverse these effects of CMS. Chronic treatment with a variety of antidepressant drugs has been reported to increase the expression and functional responsiveness of D3 receptors in the shell of the NAc (Maj et al. 1998; Lammers et al. 2000). However, the relevance of these observations for understanding the present results is uncertain, because these studies were conducted in “normal” animals (i.e., not expressing a depression-like phenotype), and because NOR was unaffected by D3 manipulations in the NAc.

In the striatum and NAc, D3 receptor expression is controlled by brain-derived neurotrophic factor (BDNF) (Guillin et al. 2001). This is potentially relevant because BDNF in the HPC is known to play a central role in mediating the behavioral effects of CMS and their reversal by chronic antidepressant drug treatment (Willner et al. 2013). However, these effects are of little help in understanding the present data, because while CMS abolished the effect of CMS on the facilitation of NOR by D3 antagonism in the HPC, antidepressant treatment did not reverse this effect. Bidirectional effects—abolition of D3-agonist-stimulated NOR by CMS and rescue by chronic antidepressant treatment—were seen only in the PFC.

The two antidepressants used in this study were chosen to be as different as possible in their neurochemical actions. VFX, a high-efficacy antidepressant (Cipriani et al. 2009), is a dual NA-5HT uptake inhibitor that also inhibits DA uptake (via the NA transporter) in PFC (Weikop et al., 2004). RSP is an atypical antipsychotic, with predominant antagonist effects at DA D2/3 and 5HT2 receptors (Leysen et al. 1994), which, like other atypical antipsychotics, is effective as adjunctive therapy for treatment-resistant depression (Wright et al. 2013) and as monotherapy in bipolar disorder (Lindström et al. 2017). In common with other antidepressants, VFX increases BDNF expression in the PFC as well as the HPC (Huang et al. 2014; Czubak et al. 2009), as, also, does RSP (Yu et al. 2015). However, an explanation of the present findings requires a physiological effect that is not only common to VFX and RSP but also present in the PFC but absent in the HPC. We have been unable to identify a potential substrate for the observed effects that meets these criteria.

## Conclusions

To our knowledge, this is the first study to examine in parallel the involvement of multiple DA receptor subtypes in multiple brain regions in both early impairment and late enhancement of NOR. We assume that memory consolidation for NOR requires endogenous DA release, leading to a relatively brief memory trace, and that boosting the DA signal by means of either D1/D2 agonists or D3 antagonists (which act pre-synaptically to increase DA release) strengthens the memory trace, such that it can be detected after a longer interval (24 h under our conditions). Previous studies have focused predominantly on D1- and D3-mediated effects in the PFC. We not only confirm these effects but also demonstrate that there is a much wider involvement of DA systems in memory for NOR, including the following: D1 receptors in all of PFC, HPC, and NAc; D3 receptors in PFC and HPC; and D2 receptors in PFC (Table 2). We speculate that the different regions may contribute different elements to memory formation: for example, an attentional component in the PFC, a spatial component in the HPC, and a motivational component in the NAc. Alongside the differential involvement of the three receptor subtypes in the three regions studied, this makes for a fertile field for further investigation.

The second half of this study addressed, we believe for the first time, the involvement of D1, D2, and D3 receptors in stress-induced cognitive impairment. The results (Table 2) demonstrate that CMS impairs D2- and D3-facilitated NOR in the PFC and HPC, and that the PFC is the site at which these impairments are rescued by chronic antidepressant treatment. D2- and D3-mediated effects were not observed in the NAc; and the HPC is ruled out as a substrate for the antidepressant effects: in this region, D2-mediated facilitation of NOR appears to be non-physiological, while D3-mediated facilitation of NOR was not rescued by antidepressant treatment.

The localization of CMS and antidepressant effects to D2 and D3 receptors in the PFC is consistent with the predominant focus on the PFC as the region of interest for studies of NOR. However, it begs the question of what neuropharmacological mechanisms are responsible for localization of CMS and antidepressant effects on NOR to the PFC specifically. This study employed two antidepressant drugs, VFX and RSP, with very different mechanisms of action, but with identical functional profiles including antianhedonic and pro-cognitive components. We have previously suggested that these two functions, as well as anxiolytic effects, involve different mechanisms and can be separately regulated (Papp et al. 2016, 2017). The antianhedonic effect is common to all antidepressants tested in the CMS model, and is thought to arise from a sensitization of D2 (or D3) receptors in the NAc (Willner 1997b; Gershon et al. 2007), while, as shown here, the pro-cognitive effect is localized to the PFC. Current theorizing on the neural substrates of depression and antidepressant action focus increasingly on the PFC (e.g., Thompson et al. 2015), but most analyses consider that effects of conventional antidepressants in the PFC either arise indirectly via primary actions in the HPC (e.g., Willner et al. 2013), or target the HPC and PFC equally (e.g., Duman and Duman 2015). A predominant action in the PFC is hypothesized for the mechanism underlying the clinical efficacy of novel rapidly acting antidepressants such as ketamine (Abdallah et al. 2016; Wohleb et al. 2017). However, as noted earlier, a PFC-specific and HPC-sparing effect of a conventional antidepressant (VFX) is a challenge to explain.

The present findings implicate dopaminergic (specifically, D2- and D3-mediated) mechanisms in the PFC as a likely substrate for cognitive impairment in depression. This conclusion would be unsurprising in relation to schizophrenia, where frontal DA systems are a major focus of attention, albeit that the primary focus in this context is on D1-mediated signals (e.g., Brisch et al. 2014; Arnsten et al. 2017). However, while the PFC is considered to be the major region mediating depressive cognitions (Ernst and Paulus 2005; Mayberg 2009; Hamani et al. 2011; Willner et al. 2013), the role of the mesocortical DA innervation in depression, which was identified as potentially important in some early studies (Di Chiara et al. 1999; Jay et al. 2004), has been largely eclipsed. The present data suggest that a revival of interest in this topic may be merited.

## Electronic supplementary material

ESM 1 (DOCX 138 kb)
