# Supplementary material for: Dopaminergic mechanisms in memory consolidation and antidepressant reversal of a chronic mild stress-induced cognitive impairment`
Source: Psychopharmacology (Berl). 2017 May 31;234(17):2571–85. doi: 10.1007/s00213-017-4651-4 (PMC5548836; doi:10.1007/s00213-017-4651-4)
Supplement: Supplementary file 1 — (DOCX 138 kb) [file 213_2017_4651_MOESM1_ESM.docx]

**Supplementary online material**

**DOPAMINERGIC MECHANISMS IN MEMORY CONSOLIDATION AND ANTIDEPRESSANT REVERSAL OF A CHRONIC MILD STRESS-INDUCED COGNITIVE IMPAIRMENT**

Mariusz Papp^1^, Piotr Gruca^1^, Magdalena Lason-Tyburkiewicz^1^, Ewa Litwa^1^, Monika Niemczyk^1^, Katarzyna Tota-Glowczyk^1^ and Paul Willner^2^

^1^Institute of Pharmacology, Polish Academy of Sciences, Krakow, Poland

^2^Department of Psychology, Swansea University, Swansea, UK

Correspondence to:

Mariusz Papp:

Institute of Pharmacology, Polish Academy of Sciences, 12 Smetna Street, 31-343 Krakow, Poland

E-mail:nfpapp@cyfronet.pl

Phone:+48 12 6623352


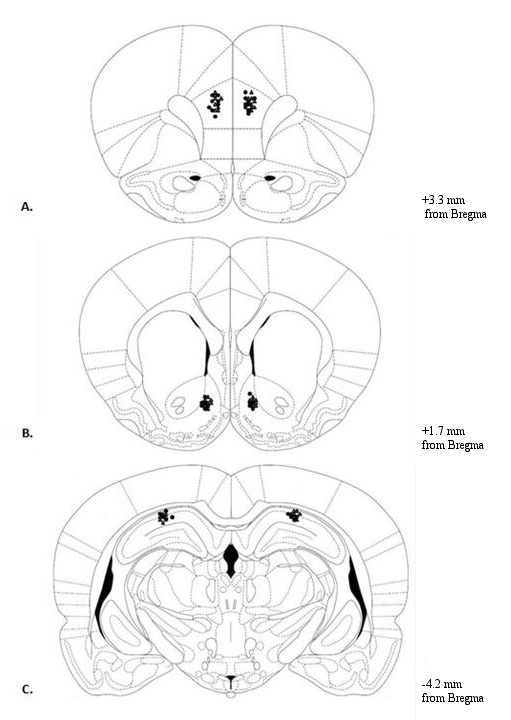


**Supplementary Figure S1:**

Schematic illustration of the sites of bilateral microinjection into (A) medial prefrontal cortex, (B) shell of nucleus accumbens and (C) CA1 of dorsal hippocampus, based on coronal sections taken from Paxinos and Watson (1997). The figure shows the location of canulla tips for saline (circles) and quinpirole (triangles) injections in experiment 2.

**Supplementary Table 1**

Statistical analyses of NOR data from experiments 1 (early impairment) and 2 (late enhancement)

|  |  | **Recognition** | | **Exploration** | | **Locomotion** | |
| --- | --- | --- | --- | --- | --- | --- | --- |
|  | df | F | p | F | p | F | p |
| **Early impairment** |  |  |  |  |  |  |  |
| ***Prefrontal cortex*** |  |  |  |  |  |  |  |
| SCH23390 | 3,28 | 4.55 | **.005** | .42 | .74 | .43 | .73 |
| L-741,626 | 2,21 | 3.73 | **.041** | .33 | .73 | 3.78 | ***.041*** |
| 7-OH-DPAT | 3,28 | 4.36 | **.012** | .38 | .77 | .79 | .51 |
| ***Hippocampus*** |  |  |  |  |  |  |  |
| SCH23390 | 3,28 | 4.40 | **.012** | 1.84 | .16 | .94 | .43 |
| L-741,626 | 3,28 | .17 | .91 | 2.22 | .11 | .64 | .60 |
| 7-OH-DPAT | 3,28 | 7.08 | **.001** | 1.08 | .37 | 1.04 | .39 |
| ***Nucleus accumbens*** |  |  |  |  |  |  |  |
| SCH23390 | 3,28 | 4.22 | **.014** | .13 | .94 | 1.33 | .29 |
| L-741,626 | 3,28 | 3.81 | ***.021*** | 1.50 | .24 | .33 | .81 |
| 7-OH-DPAT | 3,28 | .31 | .82 | 1.97 | .14 | 1.35 | .28 |
|  |  |  |  |  |  |  |  |
| **Late enhancement** |  |  |  |  |  |  |  |
| ***Prefrontal cortex*** |  |  |  |  |  |  |  |
| SKF81297 | 4,35 | 2.51 | **.059** | .53 | .71 | 2.55 | .057 |
| Quinpirole | 5,42 | 2.45 | **.049** | .32 | .90 | .33 | .90 |
| SB-277,011 | 3,28 | 4.85 | **.01** | .90 | .46 | 2.43 | .09 |
| ***Hippocampus*** |  |  |  |  |  |  |  |
| SKF81297 | 3,28 | 3.14 | **.041** | .67 | .58 | 1.04 | .39 |
| Quinpirole | 4,35 | 3.65 | **.014** | 1.57 | .21 | .80 | .54 |
| SB-277,011 | 3,28 | 2.56 | **.075** | 3.02 | ***.046*** | 1.02 | .40 |
| ***Nucleus accumbens*** |  |  |  |  |  |  |  |
| SKF81297 | 4,35 | 10.44 | **.001** | .97 | .44 | .08 | .99 |
| Quinpirole | 5,42 | .11 | .99 | .93 | .47 | 2.78 | ***.029*** |
| SB-277,011 | 3,28 | .59 | .63 | 2.03 | .13 | 1.33 | .29 |

Results of analyses of variance of drug effects at T2 (1h post-training: upper panel:) and T3 (24h post-training: lower panel). Significant effects on NOR are shown in bold. The effect of L-741,626 on NOR, shown in bold italics, was in the ‘wrong’ direction (see Fig. 2): an increase in NOR, which was not replicated when L-741,626 was re-tested at T3. Three marginally significant effects on exploration and locomotor activity are also shown in bold italics. The only significant effect relative to vehicle treatment was an increase in locomotor activity at the lower dose of L-741,626.

**Supplementary Table 2**

Statistical analyses of NOR data from experiment 3

|  | **Recognition** | | ***Planned comparisons*** | | **Exploration** | | **Locomotion** | |
| --- | --- | --- | --- | --- | --- | --- | --- | --- |
|  | F(1,34) | p | t(18), CON:  Veh vs Drug | t(18), Drug: CON vs CMS | F | p | F | p |
| ***Prefrontal cortex*** |  |  |  |  |  |  |  |  |
| SKF81297 | .01 | .92 | 3.37 **p<0.001** | .05 NS | .01 | .95 | .02 | .89 |
| Quinpirole | 2.36 | .13 | 2.88 **p<0.05** | 2.21 **p<0.05** | .89 | .35 | 1.42 | .24 |
| SB-277,011 | 1.18 | .29 | 3.18 **p<0.01** | 2.63 **p<0.05** | .10 | .75 | .15 | .70 |
| ***Hippocampus*** |  |  |  |  |  |  |  |  |
| SKF81297 | .01 | .92 | 3.32 **p<0.001** | .04 NS | .24 | .63 | .09 | .77 |
| Quinpirole | .99 | .33 | 2.19 **p<0.05** | 2.14 **p<0.05** | .14 | .71 | .03 | .87 |
| SB-277,011 | .51 | .48 | 2.58 **p<0.05** | 2.51 **p<0.05** | .52 | .48 | .14 | .71 |
| ***Nucleus accumbens*** |  |  |  |  |  |  |  |  |
| SKF81297 | 5.21 | .028 | 2.58 **p<0.05** | *2.67* ***p<0.05*** | .78 | .38 | .06 | .81 |

Results of analyses of variance of stress and drug effects at T3 (24h post-training). The F-values shown are the Stress x Challenge interaction terms. The interactions were nonsignificant but planned comparisons confirmed significant effects of all challenge drugs (confirming the results of experiment 2) and blockade by CMS of effects elicited at D2 (quinpirole) and D3 (SB-277,011) receptors, but not D1-mediated effects (SKF81297). The significant effect of CMS in the NAc is in the “wrong” direction: an increase in NOR from a low baseline. There were no significant effects on exploration or locomotor activity.

**Supplementary Table 3**

Statistical analysis of NOR data from experiment 4

|  |  | **Prefrontal Cortex** | | **Hippocampus** | |
| --- | --- | --- | --- | --- | --- |
| ***Factor (levels)*** | ***df*** | ***F*** | ***p*** | ***F*** | ***p*** |
| ***4-way anova*** |  |  |  |  |  |
| Stress (CON/CMS) | 1,120 | 32.57 | <0.001 | 13.29 | <0.001 |
| Challenge (VEH/QP/SB) | 2,120 | 54.77 | <0.001 | 25.21 | <0.001 |
| Treatment (VEH/antidepressant) | 1,120 | 10.68 | 0.001 | 2.2 | 0.14 |
| Stress x Challenge | 2,120 | 6.27 | 0.003 | 2.77 | 0.067 |
| Stress x Treatment | 1,120 | 24.92 | <0.001 | 4.28 | 0.041 |
| Challenge x Treatment | 2,120 | 3.39 | 0.037 | 3.3 | 0.041 |
| Stress x Challenge x Treatment | 2,120 | 6.1 | **0.003** | 4.16 | **0.018** |
| ***2-way anova*** |  |  |  |  |  |
| ***Quinpirole*** |  |  |  |  |  |
| Stress | 1,44 | 18.63 | <0.001 | 5.12 | 0.029 |
| Treatment | 1,44 | 6.50 | 0.014 | 8.09 | 0.007 |
| Stress x Treatment | 1,44 | 20.26 | **<0.001** | 11.63 | **<0.001** |
| ***SB-277,011*** |  |  |  |  |  |
| Stress | 1,44 | 19.11 | <0.001 | 13.42 | <0.001 |
| Treatment | 1,44 | 7.89 | 0.007 | **0.10** | **0.76** |
| Stress x Treatment | 1,44 | 12.77 | **<0.001** | **0.40** | **0.53** |

Results of analyses of variance of stress and drug effects at T3 (24h post-training). The upper half of the table shows the main effects and interactions for three of the factors in the 4-way anovas, Stress, Challenge and antidepressant Treatment. For the fourth factor, treatment drug (risperidone vs. venlafaxine) all F-values for main effects and interactions were nonsignificant (max. F=1.08). The lower half of the table shows the results of 2-way analyses conducted on each of the four drug challenges, collapsing across treatment drug and ignoring vehicle challenges. The significant interactions discussed in the text and the lack of effect of SB-277,011 in the HPC are highlighted.
